# Promising Effects of *N*-Docosahexaenoyl Ethanolamine in Breast Cancer: Molecular and Cellular Insights

**DOI:** 10.3390/molecules28093694

**Published:** 2023-04-25

**Authors:** Giuseppina Augimeri, Daniela Bonofiglio

**Affiliations:** 1 Department of Pharmacy, Health and Nutritional Sciences, University of Calabria, 87036 Rende, CS, Italy; giuseppina.augimeri@unical.it; 2 Centro Sanitario, University of Calabria, 87036 Rende, CS, Italy

**Keywords:** docosahexaenoic acid (DHA), omega-3 polyunsaturated fatty acids, breast cancer prevention, breast cancer treatment, DHA derivative, ethanolamine conjugate of DHA (DHEA), *N*-docosahexaenoyl ethanolamine

## Abstract

Unhealthy dietary habits have been identified as a risk factor for the development and progression of cancer. Therefore, adopting a healthy eating pattern is currently recommended to prevent the onset of different types of cancers, including breast carcinoma. In particular, the Mediterranean diet, based on high consumption of omega-3 polyunsaturated fatty acids (*N*-3 PUFAs), such as those found in cold-water fish and other seafood, nuts, and seeds, is recommended to reduce the incidence of several chronic-degenerative diseases. Indeed, the consumption of *N*-3 PUFAs, particularly eicosapentaenoic acid (EPA) and docosahexaenoic acid (DHA), reduced the risk of different types of cancer, including breast cancer. Moreover, they can counteract breast cancer progression and reduce the side effects of chemotherapy in breast cancer survival. Studies have demonstrated that DHA, exhibiting greater antitumor activity than EPA in breast cancer, can be attributed to its direct impact on breast cancer cells and also due to its conversion into various metabolites. *N*-docosahexaenoyl ethanolamine, DHEA, is the most studied DHA derivative for its therapeutic potential in breast cancer. In this review, we emphasize the significance of dietary habits and the consumption of *N*-3 polyunsaturated fatty acids, particularly DHA, and we describe the current knowledge on the antitumoral action of DHA and its derivative DHEA in the treatment of breast cancer.

## 1. Introduction

According to the data reported by the World Health Organization, around 30–50% of cancer cases can be prevented by decreasing exposure to risk factors associated with cancer and adopting healthy lifestyles [1]. Dietary habits have been recognized as a risk factor for several types of cancers, including breast carcinoma, which represents the most frequently diagnosed type of cancer and the primary cause of cancer-related deaths among women globally [2,3]. In the last decades, despite the fact that molecular characterizations of breast cancer can predict tumor behavior and prognosis and identify potential targets for personalized drug development and systemic therapy [4,5,6,7], metastatic disease represents the most important cause of death related to breast cancer [8]. Thus, the main goal of public health is to adopt strategies to prevent breast cancer risk. There are a variety of preventive approaches for breast cancer risk factors, among which dietary modification is currently an accepted target for breast cancer prevention [9,10]. In particular, it has been proved that a high intake of saturated and animal monounsaturated fats, which are found in red meat, cheese, and butter, has been associated with an increased risk of breast cancer, due, at least in part, to their ability to increase the levels of estrogen in the blood and supporting inflammation-driven cancer [11]. In contrast, the consumption of polyunsaturated fatty acids (PUFAs), fruits, vegetables, whole grains, and lean protein sources is linked to a reduced breast cancer risk [12,13]. PUFAs, essential fatty acids that the body cannot produce on its own and must be obtained through the diet, are divided into two classes based on the double bond of the methyl terminal, called omega-6 (*N*-6) and omega-3 (*N*-3) PUFAs. *N*-6 and *N*-3 PUFAs mediate opposite metabolic functions in the human body. In particular, *N*-3 PUFAs have anti-inflammatory and cardioprotective effects, whereas *N*-6 PUFAs promote inflammation and blood clotting. Although inflammation is a natural part of the immune response, chronic inflammation can contribute to the development of metabolic and chronic diseases such as type 2 diabetes, obesity, cardiovascular and neurodegenerative diseases, and cancer. Thus, a balanced intake of *N*-3 and *N*-6 PUFAs is recommended to maintain a healthy status [14]. In addition, several studies showed the healthy benefits of diets enriched in *N*-3 PUFAs compared to diets based on the consumption of *N*-6 PUFAs, supporting their importance in counteracting chronic-degenerative diseases. The Mediterranean diet, which suggests high consumption of fruits and vegetables, whole-grain cereals, legumes, nuts, seeds, and olive oil and moderate amounts of dairy, poultry, and red wine along with fish and seafood, is the foremost dietary pattern that promotes the consumption of *N*-3 PUFAs. Indeed, fish and other seafood, which are staples of the Mediterranean diet, are rich sources of two types of omega-3 PUFAs: eicosapentaenoic acid (EPA) and docosahexaenoic acid (DHA), whereas nuts and seeds, another key component of the Mediterranean diet, are a good source of alpha-linolenic acid (ALA), which can be converted into EPA and DHA in the body. EPA and DHA exert several positive effects in humans, including reducing inflammation, improving heart health, and preventing different types of cancer, such as breast carcinoma [15,16]. Although both *N*-3 PUFAs exert antitumoral effects in breast cancer, it has been proved that DHA has a higher antitumoral activity than EPA [17]. The reason why DHA is a more potent antitumoral compound than EPA is not well understood. It is hypothesized that its molecular structure could lead to differences in the way that this fatty acid interacts with cell membranes and signaling pathways, which could determine its stronger antitumor effects than EPA. Indeed, DHA can alter the properties of the membranes, influencing their fluidity and permeability as well as the lipid draft composition [18]. Moreover, DHA can modulate signaling proteins included in lipid microdomains, impacting the downstream signaling pathways [19]. Finally, DHA can activate different nuclear receptors than EPA influencing the expression of several genes involved in tumorigenesis [20]. Thus, based on its unique physicochemical characteristics and biochemical and physiological properties, DHA results in a very interesting molecule to be further investigated in clinical models as a potential compound for the treatment of different diseases, including breast cancer [21]. The positive effects of DHA are related to its own activity in breast cancer cells, as well as to the conversion of other metabolites. Recently, *N*-3 PUFA amides emerged as new fascinating molecules for the prevention and treatment of breast cancer [22]. These compounds, derived from the conjugation of DHA with amino acids or neurotransmitters, showed higher biological activity compared to the parental compounds, becoming promising tools for breast cancer treatment. Among the derivatives of DHA, *N*-docosahexaenoyl ethanolamine (DHEA), also called synaptamide, is the most studied molecule showing a wild spectrum of activity in various breast cancer experimental models [22,23]. The aim of this review is to highlight the importance of healthy dietary habits, specifically the consumption of *N*-3 PUFAs, and to dissect the molecular mechanism underlying the antitumor activity of DHA and its derivative DHEA in reducing the risk of breast carcinoma and potentially serving as a treatment option for breast cancer patients.

## 2. Docosahexaenoic Acid: Dietary Sources and Biosynthesis

**Figure 1:**
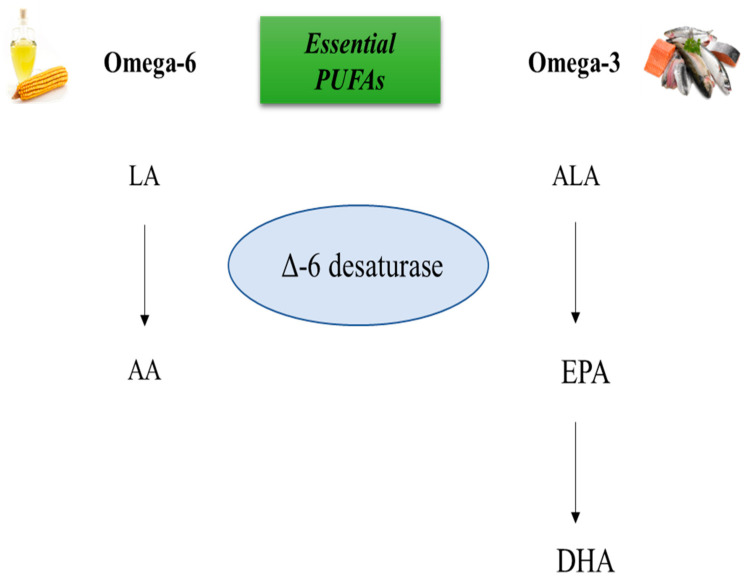
Biosynthesis of *N*-3 and *N*-6 PUFAs. The biosynthesis of *N*-3 and *N*-6 polyunsaturated fatty acids (PUFAs) involves competition between the *N*-6 PUFA linoleic acid (LA), and the *N*-3 PUFA alpha-linolenic acid (ALA), for binding to delta (∆)-6 desaturase. Depending on the amount of dietary intake of PUFAs, one of these metabolic pathways is activated. If the intake of *N*-6 PUFAs is higher than *N*-3 PUFAs, the metabolic pathway leads to the synthesis of arachidonic acid (AA). On the other hand, if the intake of *N*-3 PUFAs is higher than *N*-6 PUFAs, the metabolic pathway leads to the synthesis of eicosapentaenoic acid (EPA) and docosapentaenoic acid (DHA).

Docosahexaenoic acid, DHA, is a long-chain, highly unsaturated omega-3 fatty acid composed of 22 carbons in its acyl chain. It is characterized by the presence of six double bonds in its chain and represents the longest chain and most unsaturated fatty acid in the human body [24]. The main dietary sources of DHA are seafood, including fatty fish (tuna, mackerel, codfish, salmon, sardine, and anchovy), shellfish, micro- and macroalgae, and mother’s milk. Low amounts of DHA have been also found in meat and eggs. DHA, as well as EPA, can also be biosynthesized from its precursor, α-linolenic acid (ALA), which represents an essential fatty acid. The conversion of ALA in DHA depends on several factors, including the type of tissue and sex [25,26]. The highest conversion of ALA into DHA occurs in the liver, where the enzymes involved in this reaction, desaturase and elongase, are highly expressed. Indeed, these enzymes catalyze elongation and desaturation reactions to produce EPA from ALA into the endoplasmic reticulum. EPA can be transferred to the peroxisome and be β-oxidized to form DHA. Thus, DHA can be sent back to the endoplasmic reticulum where it can undergo esterification, lipoprotein packaging, and secretion to the blood. The *N*-3 PUFA biosynthesis pathway, called the “Sprecher pathway”, is also used by *N*-6 fatty acids, which exert important opposing physiological effects compared to *N*-3 fatty acids in the human body. In particular, the precursor of *N*-6 fatty acid, linoleic acid (LA), competes with *N*-3 ALA for access to the delta(Δ)-6 desaturase, which represents the first enzyme involved in the metabolism of PUFAs, in order to produce long-chain fatty acids [27]. The metabolic biosynthesis pathway of both PUFAs is shown in Figure 1. Thus, a balanced intake of *N*-3 and *N*-6 PUFAs is required to ensure the optimal synthesis of *N*-3 PUFAs. The ratio of *N*-6/*N*-3 varies from 1/1 to 4/1 depending on health status. In general, a low *N*-6/*N*-3 ratio is recommended to lower the risk of chronic diseases, including inflammation and cancer [28,29,30]. The recommended intake of EPA and DHA for the health of the general population is 200 to 500 mg/day EPA + DHA in the form of fish or fish oil, krill oil, or algae oil supplements. Higher amounts of EPA and DHA are suggested for the treatment of diseases including hypertriglyceridemia or inflammatory disorders such as rheumatoid arthritis [31,32]. Fish oil capsules supplemented with DHA or both DHA and EPA are safe and may cause only minor side effects, such as fishy taste, eructation, dyspepsia, diarrhea, gas, nausea, and arthralgia [33,34,35]. Recently, it has been reported that long-chain fatty acids may be beneficial for weight loss, improving appetite, body weight, post-surgical morbidity, and quality of life in cancer patients. Similarly, in a population undergoing chemotherapy and/or radiotherapy, long-chain *N*-3 fatty acids have been found to have positive effects, particularly in preserving body composition compared to control groups and protecting against chemotherapy-induced peripheral neuropathy [36].

### Biological Properties of DHA

After ingestion or biosynthesis, DHA can be stored in the membranes as esters with glycerophospholipids, conferring fluidity and selective permeability to the membranes in several tissues including the brain, heart, and retina. It has been found that 60% of the total fatty acids in the rod outer segment of the retina are composed of DHA [26], which regulates the fluidity of photoreceptor membranes, retinal integrity, and visual function [37]. Moreover, it has been demonstrated that DHA is essential for the healthy development of children’s brains, increasing the size and complexity of this tissue, and is important for the maintenance of normal brain function in adults, influencing mental, behavioral, and motor skills characterized by cognitive elements [38]. Interestingly, the brain can only convert 1% of the ALA in DHA. Thus, an adequate intake of DHA is required especially in pregnancy for the development of the growing fetal brain [39]. Apart from its role in brain development, DHA modulates inflammatory processes [40]. Indeed, it has been demonstrated that DHA reduced the production of arachnoid acid-eicosanoids and produced d-series resolvins and protectin, which exert anti-inflammatory and inflammation-resolving properties [41]. The mechanism by which DHA exerts anti-inflammatory effects has been widely investigated. It has been found that DHA reduced the production of several pro-inflammatory cytokines, including interleukin (IL)-6, tumor necrosis factor α, and IL-1β; decreased the expression of adhesion molecules; lowered the interactions between leucocytes and endothelial cells; and reduced the chemotactic response of leucocytes. Moreover, DHA can modulate the activity of key signaling pathways involved in inflammation. For example, it inhibits the nuclear factor kappa B (NF-κB) activity and enhances the expression of peroxisome proliferator-activated receptor (PPAR) γ, lowering the inflammatory cascade [41]. In the last years, it has been demonstrated that the effects of DHA are also mediated by its conversion into the acid amides, which represent an interesting class of DHA metabolites showing different biological activities to be further investigated. In particular, DHA can be conjugated with neurotransmitter or amino acids giving rise to a new class of *N*-acyl derivatives.

## 3. The Conjugate of DHA with Ethanolamine: Dietary Sources and Biosynthesis

**Figure 2:**
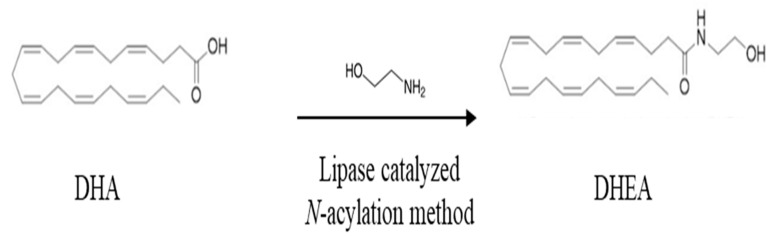
Synthesis of *N*-3 docosahexaenoic acid amide. Chemical structures of docosahexaenoic acid (DHA) and *N*-docosahexaenoyl ethanolamine (DHEA).

There are limited dietary sources of N-docosahexaenoyl ethanolamine DHEA as it is a relatively new molecule that has primarily been studied in the context of research and as a supplement. Recently, it has been revealed in the human milk of postpartum women [42]. DHEA derivatives can be synthesized from the parental compound DHA. Indeed, DHA can be derivatized with amines, alcohols, or amino acids (from the group of neurotransmitters) leading to fatty acid amides or esters showing a range of different biological activity and receptor affinity than DHA [43]. For example, fatty acid amides are divided into two subclasses: *N*-acyl amines and *N*-acyl ethanolamines. The *N*-acyl amines group includes more than 80 compounds composed of fatty acids and amino acids or neurotransmitters, such as the conjugate of DHA with dopamine, serotonin, glutamic acid and glutamine, GABA, phenylalanine, and histidine. *N*-acyl ethanolamines include the conjugates of DHA or EPA with ethanolamine, DHEA, or EPEA, respectively. The conjugate DHEA is by far the most studied fatty acid amide. As shown in Figure 2, *N*-docosahexaenoyl amine (DHEA) can be synthesized from ethanolamines and DHA through an enzymatic process that involves a direct condensation reaction between these two compounds at a molar ratio of 1:1. The reaction is carried out in *N*-hexane at a temperature of 40 °C for a duration of 15 h, and it uses the catalyst Novozym 435, which contains immobilized Candida antarctica Lipase B (Figure 2).

Based on the data reported in the literature, the formation of *N*-acyl ethanolamines depends on the intake of *N*-3 PUFAs. Animal and human studies showed that a diet enriched in *N*-3 PUFAs increases the production of the conjugates of DHA and EPA with ethanolamine. Indeed, it has been demonstrated that dietary consumption of fish oil increases the production of DHEA in the rodent brain, jejunum, liver, and adipose tissue [44,45,46,47]. Similarly, in healthy volunteers, it has been found that a daily intake of 480 mg EPA plus 360 mg DHA as fish oil food supplements doubled plasma DHEA levels in 3 weeks [43]. Moreover, DHEA has been recently detected in human milk from postpartum women and a positive correlation between DHA and DHEA concentrations has been revealed [48]. The synthesis of DHEA mainly occurs where there is local availability of DHEA precursors. For example, high concentrations of DHEA have been found in the brain and in the retina, since these tissues are characterized by the increased availability of DHA in the phospholipids. Moreover, DHEA biosynthesis can also occur from hydrolysis of the lipid *N*-acylphosphatidyl ethanolamine into *N*-acylethanolamine by the phospholipase D, whereas it can be hydrolyzed by the fatty acid amide hydrolase, FAAH, localized on the endoplasmatic reticulum [49]. Interestingly, it has been demonstrated that different cancer cell lines, including prostate and breast cancer cells, can produce DHEA upon treatment with DHA [23].

### Biological Activity of DHA with Ethanolamine

To date, little is known about the biological activity of DHEA. Although only a few studies have been carried out, DHEA seems to be a very interesting molecule showing anti-inflammatory properties. In particular, it has been demonstrated that DHEA reduced the release of nitric oxide (NO) and the secretion of the chemokine CCL2, also called Monocyte chemoattractant protein-1 (MCP-1), in lipopolysaccharide (LPS)-stimulated RAW264.7 macrophages [50] along with a decreased expression of cyclooxygenase (COX-2) and COX-2-derived eicosanoids [51]. Moreover, it has been shown that DHEA reduced the production of CCL2, IL6, and NO in mouse peritoneal macrophages. Finally, the anti-inflammatory effects of DHEA have been also revealed in differentiated 3T3-L1 adipocytes, where DHEA was able to reduce the production of CCL2 and IL6 induced by the treatment with LPS [52]. In addition to the anti-inflammatory properties, both DHA and its precursor compound, DHEA, play a crucial role in brain development and the preservation of cognitive function. It has been shown that DHEA stimulated neurite growth and synaptogenesis and enhanced glutamatergic synaptic activity in hippocampal neurons [43,53,54]. Interestingly, the metabolites of DHEA showed biological activity. Indeed, they are substrates for COXs, lipoxygenases (LOXs), and cytochrome P450 enzymes, modulating the inflammatory response. Moreover, DHEA is converted into oxygenated molecules, such as 17-hydroxy-DHEA, 10,17-dihydroxy-DHEA, and 15-hydroxy-16(17)-epoxy-DHEA, which showed anti-inflammatory properties [55].

## 4. Antitumoral Effects of Docosahexaenoic Acid in Breast Cancer

The effects of DHA have been widely investigated in preclinical and clinical studies of breast cancer. In vitro studies have shown that DHA-induced apoptosis in different breast cancer cells either alone or in combination with chemotherapeutic drugs through several mechanisms that are not fully understood [56,57]. To date, it has been demonstrated that the incorporation of DHA into the cell membrane is required for the activation of the apoptosis cascade in breast cancer cells. In triple-negative MDA-MB-231 breast cancer cells, DHA led to modifications in the biophysical characteristics of lipid rafts. This, in turn, resulted in a reduction of breast cancer cell proliferation by decreasing the cholesterol content and potentially altering the distribution of crucial proteins in the lipid rafts [58]. In addition, in the HB4aC5.2 cell normal breast cell line overexpressing HER2, it has been observed that DHA treatment disrupted lipid raft, and inhibited HER2 signaling, enhancing apoptosis [59]. The mechanism by which the change in DHA content of membrane-induced breast cancer cell apoptosis is still under investigation. Several routes are currently taking place, including the increase in intracellular oxidative stress, lipid peroxidation, and the modulation of eicosanoid metabolites [60]. Interestingly, it is known that DHA modulates the activation of nuclear receptors, leading to a change in the expression of the genes involved in tumorigenesis. Among the transcription factors regulated by DHA, the most important are NF-κB (70), activator protein 1 (AP-1) (71,72), c-myc (10,73), p53 (74,75), and PPARs. Another mechanism by which DHA reduced breast cancer cell proliferation is related to its ability to modulate the expression of cell-cycle molecules, thus inducing arrest in the G1 phase and G2M phase of the cell cycle [61,62,63,64,65]. DHA has been shown to downregulate the expression of cyclin D1 and cyclin-dependent kinase (CDK)4/6, which are required for the G1/S transition of the cell cycle. Moreover, in MCF-7 breast cancer cells, it has been found that DHA reduced cell proliferation through proteasome-dependent degradation of the ERα, as well as a decrease in the expression of the cyclin D1 and inhibiting the mitogen-activated protein kinase MAPK signaling. Additionally, DHA has been shown to upregulate the expression of CDK inhibitors, including p21 and p27, which promote cell-cycle arrest in the G1 and G2/M phases. Recently, it has been demonstrated that DHA can trigger pyroptosis, a programmed process mediated by proteases so-called inflammatory caspases, increasing the activation of caspase-1 and gasdermin D, as well as increasing the secretion of IL-1β, which results in the membrane ore formation and cell death [66]. The effects of DHA in breast cancer treatment have been investigated not only in preclinical studies but also in animal models. However, most of the in vivo studies have investigated the action of a mixture of *N*-3 PUFAs, particularly DHA and EPA, in counteracting breast cancer development. Moreover, only a few studies have evaluated the action of DHA in the treatment of established tumors, whereas the role of DHA in preventing breast cancer development has been widely investigated. For example, it has been reported that increasing the ratio of *N*-3 PUFA to *N*-6 PUFA reduced mammary cancer incidence.

Interestingly, it has been observed that DHA is a chemosensitizer agent in different types of cancer, including breast cancer. In MDA-MB-231 breast cancer cells, it has been demonstrated that DHA increased the sensibility of several chemotherapeutic drugs, including paclitaxel, doxorubicin, and docetaxel [67,68,69]. Moreover, it has been demonstrated that DHA administration reduced the tumor vascular density in rats treated with epirubicin [70]. The mechanism by which DHA enhanced the effects of chemotherapeutic molecules is not fully understood. However, it has been demonstrated that the chemosensitizer effects of DHA are related, at least in part, to its ability to increase the peroxidative processes and modulate the oncoprotein expression [71]. Moreover, DHA showed higher chemosensitizer properties when it was administered in combination with EPA. For example, supplementation with DHA alone did not enhance the efficacy of 5-fluorouracil, cyclophosphamide, and gemcitabine in mouse models of colon cancer, supporting the concept that a mixture of *N*-3 PUFAs is more effective than single supplementation of DHA or EPA.

Clinical studies have, to date, been variable results on the benefits of PUFAs in breast cancer. For example, a clinical trial investigating the effects of *N*-3 PUFA supplementation with raloxifene in postmenopausal women did not show changes in secondary endpoint blood risk biomarkers, whereas a favorable modulation of different tissue risk markers has been observed for breast cancer in premenopausal and postmenopausal women [35,72]. In contrast, positive results have been obtained in breast cancer patient survival. Indeed, a reduction in breast cancer recurrence and an improvement in overall mortality has been found in women with early-stage breast cancer followed for a median of 7 years [73]. Moreover, DHA was shown to ameliorate the outcome of metastatic breast cancer patients in a phase II trial [74]. To date, *N*-3 PUFAs are considered immunonutrients to be prescribed to cancer patients along with glutamine, arginine, and ribonucleotides to maintain immunocompetence during the chemotherapeutic treatment. Moreover, *N*-3 PUFAs represent pharmaconutrients used to reduce treatment-related complications, including cancer-related pain, anorexia-cachexia syndrome, and depression disorder [75]. These results underscore the significance of taking into account the interaction between diet and chemotherapeutic drugs to optimize the efficacy of pharmacological treatments, reduce the risk of recurrence, and minimize treatment-related toxicity and mortality. However, our comprehension of this interaction remains limited, and more extensive research is required to explore this area in greater depth. As the current studies have been conducted on relatively small patient populations, there is a pressing need for larger and more comprehensive studies. Consequently, various organizations have issued dietary recommendations, including guidelines for omega-3 dietary intake [76].

In the last years, different preclinical and clinical studies showed that DHA conjugate with chemotherapeutic drugs has a strong antitumoral effect used as a single agent, reducing the side effects of multiple drug therapy. In particular, it has been found that DHA conjugated with gemcitabine displayed a stronger activity than gemcitabine alone, reducing breast cancer tumor growth [77]. In addition, DHA conjugated with paclitaxel reduced the side effects induced by paclitaxel treatment in patients with resistant solid tumors, including breast cancer patients [78]. In vitro experiments have also shown that DHA conjugated with propofol reduced breast cancer cell migration and increased apoptosis [79]. Recently, it has been demonstrated that the codelivery of doxorubicin, DHA, and α-tocopherol succinate by nanostructured lipid carriers displayed synergistically antitumor effects in both breast cancer cells and mice models, decreasing doxorubicin-induced toxicity in animals [80]. Thus, these codelivery nanosystems improving therapeutic efficiency may be considered promising tools to be used for breast cancer treatment.

## 5. Antitumoral Effects of DHEA in Breast Cancer

Since breast cancer cells have the ability to convert DHA into DHEA, exerting a higher antitumoral activity, several studies have investigated the effects of this compound in breast cancer. According to the data presented in the literature, DHEA has higher antiproliferative effects than DHA in breast cancer cells while exhibiting no adverse impact on the viability of non-tumorigenic mammary epithelial cells [81]. These observations shed light on DHEA as a promising candidate for the treatment of breast cancer. However, the research is still far from a conclusion and additional investigations are required to suggest this molecule as a potential drug for breast cancer. In estrogen receptor α-positive breast cancer cells, Rovito et al., have demonstrated that DHEA inhibited cancer cell proliferation in a concentration-dependent manner, reaching an IC_50_ value of 0.8 µM after 96 h in MCF-7 cells [82]. The antiproliferative effects of DHEA in MCF-7 breast cancer cells are mediated by the activation of the PPARγ, a nuclear receptor involved in several diseases, such as inflammation, metabolic disorders, cardiovascular disease, and different types of cancer [83,84]. In particular, it has been documented that activation of PPARγ by agonists induced breast cancer cell death and inhibits breast cancer progression [85]. Rovito et al., have shown that DHEA, acting as a ligand, upregulated PPARγ mRNA and protein levels as well as the expression of the PPARγ target gene phosphatase and tensin homolog on chromosome ten (PTEN). Interestingly, the enhanced PTEN protein levels were associated with the decrease in phosphoinositide 3 kinase (PI3K)/Akt/mammalian (or mechanistic) target of the rapamycin (mTOR) signaling pathway. The PI3K/Akt/mTOR signaling pathway is one of the most common dysregulated signaling pathways in breast cancer, leading to increased cell proliferation and survival. This dysregulation can occur through multiple mechanisms, including activating mutations in PI3K or loss of negative regulators, such as the tumor suppressor PTEN. DHEA not only decreases the activation of the PI3K/Akt/mTOR signaling pathway but also increases the expression of Beclin-1, which triggers autophagy of breast cancer cells enhancing the formation of the autophagosome. Specifically, Beclin-1 binds to the Bcl-2 homology-3 (BH3) domain, resulting in the inhibition of Beclin-1 autophagic function. It has been demonstrated that DHEA promotes autophagosome formation by increasing the phosphorylation of Bcl-2 at serine 70, thereby reducing its physical interaction with Beclin-1 and resulting in elevated levels of the unbound Beclin-1 protein [82]. DHEA can exert antiproliferative effects in breast cancer cells not only through the activation of PPARγ but also by binding to the cannabinoid receptors (CBs) [86]. Brown et al., have demonstrated that the antiproliferative effects of DHEA in ERα positive MCF-7 and triple-negative MDA-MB-231 breast cancer cells are, at least in part, mediated by its binding to the CB1, since the inhibition of the CB1 by specific antagonists decreased the antiproliferative and antitumoral activities of DHEA in breast cancer cells. The effects of DHEA in reducing cell proliferation involve the MAPK signaling pathway. It has been widely demonstrated that the activation of the MAPK signaling pathway promotes cancer cell proliferation by inducing cell-cycle progression, increasing cell survival, and inhibiting apoptosis. Moreover, it can also promote cancer cell migration and invasion by regulating cytoskeletal dynamics and modulating the expression of genes involved in epithelial-mesenchymal transition. The authors found that DHEA treatment decreases the expression of p38 MAPK and phosphorylated p38 MAPK (pp38) as well as the phosphorylated ERK, thus impacting breast cancer proliferation [81]. Recently, the effects of DHEA in triple-negative breast cancer cells have been investigated, demonstrating its potential antitumoral action [87]. In particular, it has been reported that DHEA reduced the viability of both MDA-MB-231 and MDA-MB-436 breast cancer cells in a concentration-dependent manner, showing IC_50_ values of 27.29 μM and 19.76 μM, respectively. Moreover, nontoxic concentrations of DHEA shifted MDA-MB-231 and MDA-MB-436 cells from an energetic to a quiescent state. Interestingly, DHEA affected cell motility and invasiveness of triple-negative breast cancer cells, which represent two distinct characteristics of this aggressive breast cancer subtype. These results are partially in agreement with the data published by Brown et al., who showed that DHEA reduces cell invasion without affecting cell migration [81]. To better understand the mechanism of action of DHEA in triple-negative breast cancer cells, Augimeri et al., analyzed the breast cancer cell secretome upon DHEA treatment [87]. Indeed, the role of secreted factors in mediating the phenotype of cancer cells as well as their interaction with the cells of the tumor microenvironment has been widely demonstrated. DHEA treatment has modified the secretion of 28 molecules interconnected in a protein–protein network. Among these proteins, we observed that the C-C motif chemokine ligand 5 (CCL5) represented a key regulator factor and mediated the effect of DHEA in reducing the motility of triple-negative breast cancer cells. Moreover, the authors have demonstrated that DHEA, by decreasing CCL5 secretion, affected the inflammatory tumor microenvironment, reducing macrophage recruitment. In addition, DHEA reduced macrophage viability as well as the expression of tumor-associated macrophage (TAM) markers in a paracrine fashion. Interestingly, DHEA can also affect the macrophage phenotype through a direct effect on these cells. Indeed, Gionfriddo et al., have demonstrated that DHEA attenuated the secretion of cytokines associated with the M1 and M2 macrophage phenotype in a PPARγ-dependent manner in breast TAMs [88].

**Figure 3:**
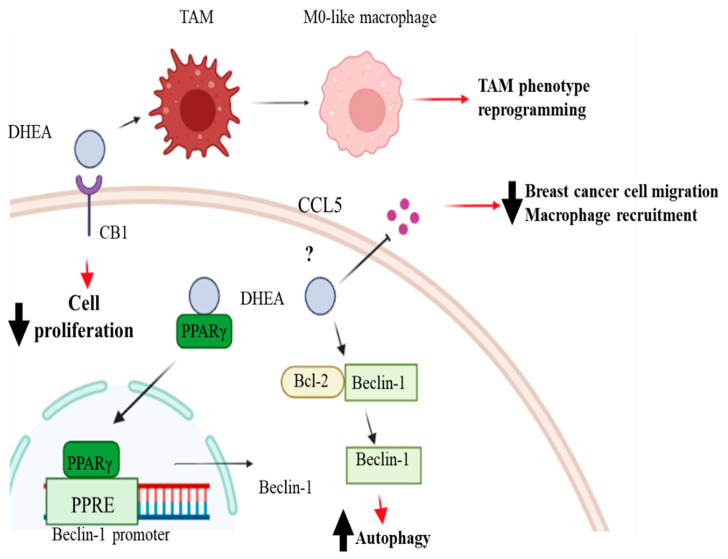
Mechanism of action and biological effects of DHEA in breast cancer. Docosahexaenoyl ethanolamide (DHEA) can increase Beclin-1 expression through the peroxisome proliferator-activated receptor (PPAR) γ binding to its response element (PPRE) in the promoter region of Beclin-1 and increase its dissociation from the Beclin-1/Bcl2 complex, inducing autophagy. DHEA can also bind to cannabinoid receptor 1 (CB1), which partially mediates its antiproliferative and antitumoral effects in breast cancer cells. DHEA can also modify the secretion of the C-C motif chemokine ligand 5 (CCL5), reducing breast cancer cell migration and macrophage recruitment. Furthermore, DHEA can affect the inflammatory tumor microenvironment, affecting tumor-associated macrophage (TAM) markers’ expression, and reprogramming the TAM phenotype. Created with BioRender.com.

The specific mechanisms involved in DHEA antineoplastic effects in breast cancer cells are summarized in Figure 3.

To date, the effects of DHEA in combination with chemotherapeutic drugs are still unknown. Moreover, despite extensive research into the favorable effects of DHEA in mitigating tumor aggressiveness, there is currently a lack of animal and clinical studies investigating this phenomenon.

## 6. Conclusions

Adopting a healthy eating pattern, such as the Mediterranean diet, that includes high consumption of *N*-3 PUFAs, particularly DHA, is recommended for the prevention and treatment of breast cancer. However, more research is necessary to fully understand the mechanism of action of DHA, particularly the *N*-docosahexaenoyl ethanolamine for breast cancer. While intriguing data suggest the antitumor effects of DHEA in breast cancer cells, its effect in in vivo models needs to be further investigated. Moreover, until now the potential beneficial effect of the combination of chemotherapy with supplementation/treatment with DHEA is not well recognized. Therefore, in our opinion, more attention should be paid to the application of DHEA in the prevention and treatment of breast cancer.

## Data Availability

Data sharing is not applicable.
